# Anti-inflammatory and immune regulatory effects of acupuncture after craniotomy: study protocol for a parallel-group randomized controlled trial

**DOI:** 10.1186/s13063-016-1712-7

**Published:** 2017-01-10

**Authors:** Seung-Yeon Cho, Seung-Bo Yang, Hee Sup Shin, Seung Hwan Lee, Jun Seok Koh, Seungwon Kwon, Woo-Sang Jung, Sang-Kwan Moon, Jung-Mi Park, Chang-Nam Ko, Seong-Uk Park

**Affiliations:** 1 Department of Cardiology and Neurology, College of Korean Medicine, Kyung Hee University, 26, Kyungheedae-ro, Dongdaemun-gu, Seoul 02447 Republic of Korea; 2 Department of Clinical Korean Medicine, Graduate School, Kyung Hee University, 26, Kyungheedae-ro, Dongdaemun-gu, Seoul 02447 Republic of Korea; 3 Department of Neurosurgery, College of Medicine, Kyung Hee University, 26, Kyungheedae-ro, Dongdaemun-gu, Seoul 02447 Republic of Korea; 4 Stroke and Neurological Disorders Center, Kyung Hee University Hospital at Gangdong, 892, Dongnam-ro, Gangdong-gu, Seoul 05278 Republic of Korea

**Keywords:** Craniotomy, Neurosurgery, Acupuncture, Electroacupuncture, Inflammation, Immune function

## Abstract

**Background:**

Despite recent advances in the medical and surgical fields, complications such as infection, pneumonia, or brain swelling may occur after a craniotomy. In some patients, perioperative antibiotic prophylaxis causes adverse effects such as itching, rash, or digestive conditions. Certain patients still develop infections severe enough to require a repeat operation despite antibiotic prophylaxis. Acupuncture has been used to treat inflammatory conditions, and many basic and clinical studies have provided evidence of its anti-inflammatory and immune regulatory effects. The aim of this study is to explore the effects of acupuncture on inflammation and immune function after craniotomy.

**Methods:**

This trial will be a single-center, parallel-group clinical trial. Forty patients who underwent craniotomy for an unruptured aneurysm, facial spasm, or a brain tumor will be allocated to either the study or the control group. The study group will receive conventional management as well as acupuncture, electroacupuncture, and intradermal acupuncture, which will start within 48 h of the craniotomy. The patients will receive a total of six sessions within 8 days. The control group will only receive conventional management. The primary outcome measure will be the C-reactive protein levels, while the secondary outcomes will be the serum erythrocyte sedimentation rate and the tumor necrosis factor-α, interleukin (IL)-1β, and IL-6 levels measured at four different time points: within 48 h prior to the craniotomy and on days 2, 4, and 7 after surgery. The presence of fever and infection, the use of additional antibiotics, the presence of infection, including pneumonia or urinary tract infection, and safety will also be investigated.

**Discussion:**

In this trial, we will observe whether acupuncture has anti-inflammatory and immune regulatory effects after a craniotomy. If our study yields positive results and a placebo-controlled study also finds favorable results following our study, acupuncture could be recommended as an adjunctive therapy after a craniotomy.

**Trial registration:**

ClinicalTrials.gov: NCT02761096. Registered on 27 April 2016.

**Electronic supplementary material:**

The online version of this article (doi:10.1186/s13063-016-1712-7) contains supplementary material, which is available to authorized users.

## Background

A craniotomy is a common surgical procedure performed to remove brain tumors, blood, or arteriovenous malformations (AVM), and to clip an aneurysm, or for microvascular decompression (MVD). Even if the surgery is performed properly, complications, such as infection, pneumonia, or brain swelling, may occur after a craniotomy [1]. Infections are one of the most common postoperative complications, and impairment of immune function after surgery has been shown to be associated with a higher risk of infection as well as other postoperative complications [2]. C-reactive protein (CRP) is an indicator of inflammatory processes and its levels rise in response to surgical procedures or infection. Therefore, it is commonly used as an index of complications after surgery [3–7]. A study that investigated the association between changes in serum proinflammatory and anti-inflammatory cytokine concentrations and postoperative septic complications reported that the postoperative increase in interleukin (IL)-6 concentration was associated with septic morbidity, while an elevated level of the IL-1 receptor antagonist (IL-1ra) was associated with postoperative septic shock [8].

Appropriate antibiotic prophylaxis reduces the incidence of inflammation, although it may cause adverse effects such as itching, rash, digestive problems including diarrhea, or elevation of liver enzyme levels in some patients. Despite recent advances in the medical and surgical fields that have improved postoperative results, certain patients continue to develop severe infections and require a repeat operation, which is associated with increased morbidity and mortality [9, 10]. Therefore, methods that are complementary to conventional postoperative treatment for reducing inflammation and regulating immune function are needed.

Acupuncture has been used to treat a variety of inflammatory conditions including asthma, rhinitis, inflammatory bowel disease, and rheumatoid arthritis. Growing evidence indicates that acupuncture significantly inhibits the inflammatory response [11]. Findings from several clinical studies support the anti-inflammatory effects or immunological functions of acupuncture. Patients with acute pancreatitis, who received 7 days of electroacupuncture, had higher serum IL-10 and lower CRP levels [12]. Based on a systematic review by McDonald et al., reductions in the erythrocyte sedimentation rate (ESR) and CRP levels were observed after patients with rheumatoid arthritis received acupuncture [13]. It has been shown that the anti-inflammatory effects of acupuncture are mediated by downregulation of proinflammatory cytokines such as tumor necrosis factor-alpha (TNF-α), IL-1β, IL-6, and IL-10 [14]. Elevated plasma TNF-α levels in rats exposed to endotoxin decreased after electroacupuncture at *Zusanli* (ST36) [15]. In rats with chronic obstructive pulmonary disease (COPD), TNF-α and IL-1β levels in the bronchoalveolar lavage fluid decreased after electroacupuncture [16]. In rats with spastic cerebral palsy, serum TNF-α, IL-6, CRP, and nitric oxide synthase levels significantly decreased in the acupuncture group [17]. In experimental rats with periodontitis, electroacupuncture was seen to modulate the immune inflammatory response by decreasing the expression of IL-1β and matrix metalloproteinase-8, and increasing IL-6 messenger ribonucleic acid (mRNA) expression [18]. In addition, several studies have been conducted to evaluate the anti-inflammatory effects and regulation of immunity by acupuncture [19, 20].

However, few acupuncture studies focus on its anti-inflammatory effects and effects on immunity in patients who have undergone surgery. In a study with thyroidectomy patients, the plasma CRP levels at the time of surgery and on days 1 and 3 after surgery were remarkably lower in the electroacupuncture group compared to the sham group [21]. In a study of the immune-inflammatory response of patients undergoing supratentorial craniotomy, the levels of IL-10 and IL-8 significantly increased, while the TNF-α, IgM, and IgA levels also changed significantly after electroacupuncture [2]. However, the effects of acupuncture on the anti-inflammatory response after a craniotomy have not been identified. The decrease in the inflammatory response and regulation of immune function are important for rapid recovery from, and prevention of, complications after surgery. The aim of this study is to explore the anti-inflammatory and immune regulatory functions of acupuncture after a craniotomy.

## Methods

**Fig. 1:**
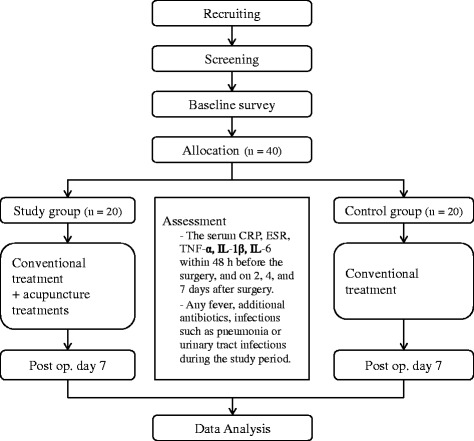
Study protocol flow chart

This study will be a single-center, parallel-group clinical trial that will be conducted at the Kyung Hee University Hospital at Gangdong, Seoul, Korea. The flow chart of the trial is shown in Fig. 1. The Standard Protocol Items: Recommendations for Interventional Trials (SPIRIT) 2013 Checklist is given in Additional file 1.

### Ethics

The trial will be carried out in accordance with the Declaration of Helsinki and the Korean Good Clinical Practice Guidelines and has been approved by the Ethical Committee of the Kyung Hee University Hospital at Gangdong (KHNMC-OH-IRB 2016-01-005). It has been registered at https://clinicaltrials.gov/ (NCT02761096).

### Participants

#### Subject enrollment and allocation

A total of 40 participants will be recruited for this trial. Patients who underwent a craniotomy for an unruptured aneurysm, facial spasm, or brain tumor are potential candidates for the study. After written informed consent has been obtained, eligible participants will be allocated to the study group or the control group. Patients who agree to receive additional acupuncture treatment will be allocated to the study group, while patients who do not agree will be allocated to the control group.

#### Inclusion criteria

Participants must meet all of the following criteria in order to be included: (1) planning to undergo regular craniotomy performed for an unruptured aneurysm, facial spasm, or brain tumor, (2) be aged over 18 years, (3) agree to acupuncture treatment that can start within 48 h after the craniotomy, and (4) voluntary participation and provision of a signed Informed Consent Form.

#### Exclusion criteria

Participants with any of the following conditions will be excluded: (1) serum CRP level ≥ 1.0 mg/dl before the craniotomy, (2) a condition other than an unruptured aneurysm, facial spasm, or brain tumor as an indication for craniotomy, (3) a craniotomy performed for infectious brain diseases such as brain abscess or subdural empyema, (4) medication use that can affect the immune system or white blood cell (WBC) count, such as immunosuppressive drugs, steroids, or anticancer drugs or use of these medications within 1 month prior to the craniotomy, (5) a history of surgery at the same site, (6) undergoing emergency surgery, (7) a severe medical disease, e.g., congestive heart failure, chronic renal failure or an autoimmune disorder, (8) a pacemaker or an implantable cardioverter defibrillator, or (9) pregnancy.

### Intervention

#### Study group

The subjects in the study group will receive acupuncture treatments in addition to conventional treatment before and after the craniotomy. The conventional treatment involves general management in the Department of Neurosurgery and includes stabilizing vital signs, pain control, perioperative antibiotic prophylaxis, treatment of infections, and other intravenously administered fluid or drug therapy required based on the condition of the patient.

The acupuncture intervention will start no more than 48 h after the craniotomy and will be administered once a day for 6 days (a total of six sessions within 8 days). It will be given in addition to conventional treatments. All interventions will be performed by one Korean Medicine doctor with over 5 years of working experience and a college education of 6 years. This doctor will be trained in the study protocol before the start of the trial.

Acupuncture, electroacupuncture, and intradermal acupuncture will be performed during every session. Sterile disposable stainless steel acupuncture needles (0.25 mm × 30 mm; Dong Bang Acupuncture Inc., Chungnam, Korea) will be used. Acupuncture needles will be inserted bilaterally at the following acupuncture points: LI4, LI11, PC6, ST36, GB39 and LR3, and GV20 [12, 15, 18, 21]. If the GV20 is close to the surgical site, this acupoint may be excluded. After insertion to a depth of approximately 0.5–1.5 cm, the needles will be manually stimulated until *de qi*, which is a subjective experience in which patients feel a radiating sensation considered indicative of effective needling, is achieved.

An electric stimulator (ES-160, ITO Co., Tokyo, Japan) will be connected to the handle of each needle at LI4, LI11, ST36, and LR3 and a current of 5 Hz will be applied. The current intensity will be increased until light muscle contraction is evident and reaches approximately 70% of the bearable intensity. The needles will be left in place for 15 min and then removed. The practitioner will be able to regulate the intensity in response to requests from the patients.

After the needles are removed, intradermal acupuncture needles with tape (DB130A; 0.25 mm × 1.5 mm; Dong Bang Acupuncture Inc., Chungnam, Korea) will be inserted at the same acupoints (LI4, LI11, PC6, ST36, GB39, and LR3 bilaterally, and GV20) and left in place until the next session.

#### Control group

The subjects in the control group will receive conventional treatment alone before and after the craniotomy in the Department of Neurosurgery.

### Assessment

The serum CRP level, ESR, TNF-α, IL-1β, and IL-6 levels will be assessed four times: within 48 h before the surgery, and 2, 4, and 7 days after surgery. A fasting blood sample (8 ml) will be drawn from the brachial vein at a fixed time in the morning before breakfast, centrifuged immediately, and kept in a freezer at −80 °C before the analysis.

**Fig. 2:**
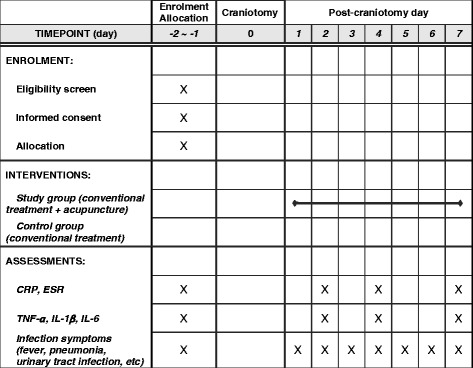
The schedule of enrollment, interventions, and assessments

Any fever with a body temperature over 38 °C, the use of additional antibiotics, and infections, such as pneumonia or urinary tract infections, will be recorded on the Case Report Form every day during the study period (Fig. 2).

#### Dropout criteria

Participants who meet any of the following criteria will be excluded from the study: (1) more than one session (out of a total of six) missed, (2) need for a repeat operation or other types of surgery, (3) serious neurological deficits that develop after the surgery and significantly worsen level of consciousness and motor skills compared with the preoperative condition, (4) nonconventional antibiotics received immediately after surgery, (5) withdrawal of consent, (6) development of serious adverse reactions and inability to continue the trial, (7) worsening condition whereby it is no longer appropriate for the patient to continue to participate in the study (as decided by the investigator), or (8) a decision by the principal investigator that it is not possible for the patient to participate in the study as planned.

### Outcome measures

#### Primary outcome measurement

The change in the serum CRP level from baseline to the second day after surgery will be compared between the two groups. CRP is a known indicator of inflammation and is frequently used to support the diagnosis of an inflammatory process. It has been found to be a better marker of an acute phase reaction than ESR [22].

#### Secondary outcome measurement

The secondary outcome measures are as follows:

1. The difference in CRP level changes (pre versus post) between the two groups
2. Serum ESR: ESR is surrogate marker of an acute phase inflammatory reaction [22]
3. TNF-α, IL-1β, and IL-6: TNF-α, IL-1β, and IL-6 are cytokines that are related to inflammation and immune regulation [23–25]. After samples are collected, they will be centrifuged for 15 min at 1000 × g and stored in a freezer at −80 °C until they are ready for processing and analysis. The Quantikine® ELISA kit (R&D systems, Inc., Minneapolis, MN, USA) will be used to determine the concentrations of TNF-α, IL-1β, and IL-6 in the samples and the data will be analyzed. All the samples will be discarded after the analysis.
4. The number of days for which the patient had a body temperature greater than 38 °C
5. Antibiotic use in addition to the conventional antibiotics prescribed after the surgery
6. Pneumonia diagnosed by symptoms such as fever, cough or sputum production, findings on a chest radiograph, or sputum culture
7. Urinary tract infection diagnosed by symptoms such as fever, dysuria, increased urinary frequency with chills, and from results of the urine analysis
8. Other symptoms and findings consistent with an infection

### Safety evaluation

Any adverse events or abnormalities will be recorded on the Case Report Forms. Severity will be quantified as mild, moderate, or severe. The occurrence of events during or after the intervention will be categorized as unrelated, possibly related, or related.

If any serious adverse events occur, the study will be stopped immediately and appropriate action will be taken. This will be reported promptly to the Institutional Review Board in accordance with the protocol.

### Sample size estimation

The primary outcome, the change in serum CRP level from baseline to the second day after surgery, was used to calculate the sample size. Power analysis indicated that a sample size of 16 subjects per group will be required to detect a serum CRP difference of 3.2 mg/dl with a standard deviation of 3.190 with 80% power and a significance level of 5% in a two-tailed (or two-sided) *t* test. We plan to enroll a total of 40 participants with 20 in the study group and 20 in the control group allowing for a 20% withdrawal rate. The sample size was calculated using the followig formula [26]:

$$ n=\frac{2{\left({z}_{a/2}+{z}_{\beta}\right)}^2{\sigma}^2}{{\left({\mu}_c-{\mu}_t\right)}^2} $$

### Statistical analyses

The statistical analyses will be performed by a researcher who is blinded to the allocation. Following the per-protocol (PP) principle, all the data will be analyzed using SPSS software (version 18.0, SPSS Inc., Chicago, IL, USA) and the results will be presented as mean ± standard deviation (SD) or number (%).

In order to compare the change in serum CRP levels from baseline to the second day after surgery, the average number of days that fever occurred and the number of additional antibiotics used between the two groups, either a *t* test or a Mann-Whitney *U* test will be used. The differences in pre and postoperative blood test results between the two groups will be compared using a generalized linear mixed model (GLMM). To compare events due to infections such as pneumonia, urinary tract infection or other diseases, either the chi^2^-test or the Fisher’s exact test will be used. If the data are not normally distributed, nonparametric methods will be used. Confidence limits of 95% will be calculated, and the results with a *p* value < 0.05 will be considered statistically significant.

## Discussion

In this study, we aim to explore the possibility that treatment with acupuncture after a craniotomy has anti-inflammatory and immune regulatory effects. Subjects will be assigned to either the study group or the control group on a voluntary basis. If our results suggest efficacy and if a placebo-controlled study also yields favorable results, acupuncture could be recommended as an adjunctive therapy after a craniotomy as it could possibly reduce inflammatory and immune responses after a craniotomy.

### Trial status

The recruitment started in May 2016 and will be completed by the end of March 2017.

## Additional file

Additional file 1: SPIRIT Checklist. (DOCX 60 kb)

## Abbreviations

CRP: C-reactive protein
ESR: Erythrocyte sedimentation rate
IL: Interleukin
TNF-α: Tumor necrosis factor-alpha
