# Correction to: Anti-inflammatory and immune regulatory effects of acupuncture after craniotomy: study protocol for a parallel-group randomized controlled trial

**DOI:** 10.1186/s13063-017-2230-y

**Published:** 2017-10-16

**Authors:** Seung-Yeon Cho, Seung-Bo Yang, Hee Sup Shin, Seung Hwan Lee, Jun Seok Koh, Seungwon Kwon, Woo-Sang Jung, Sang-Kwan Moon, Jung-Mi Park, Chang-Nam Ko, Seong-Uk Park

**Affiliations:** 1 Department of Cardiology and Neurology, College of Korean Medicine, Kyung Hee University, 26, Kyungheedae-ro, Dongdaemun-gu, Seoul, 02447 Republic of Korea; 2 Department of Clinical Korean Medicine, Graduate School, Kyung Hee University, 26, Kyungheedae-ro, Dongdaemun-gu, Seoul, 02447 Republic of Korea; 3 Department of Neurosurgery, College of Medicine, Kyung Hee University, 26, Kyungheedae-ro, Dongdaemun-gu, Seoul, 02447 Republic of Korea; 4 Stroke and Neurological Disorders Center, Kyung Hee University Hospital at Gangdong, 892, Dongnam-ro, Gangdong-gu, Seoul, 05278 Republic of Korea

## Correction

In the original publication [1] was one author affiliation of the author Seung-Bo Yang incorrect. Only affiliation 2 belongs to Seung-Bo Yang.

The correct affiliations can be found below:

Seung-Bo Yang.

Department of Clinical Korean Medicine, Graduate School, Kyung Hee University, 26,
